# Transcending the Corporeal in Later Old Age?

**DOI:** 10.1093/geroni/igab046.2263

**Published:** 2021-12-17

**Authors:** Paul Higgs

## Abstract

This symposium addresses the older body and later life. It focusses on the cultural and social implications of the corporeality of the ageing body. Specifically it seeks to explore the degree to which it is possible to transcend the constraints brought about by the body in later old age. Drawing the distinction between the third and fourth ages for understanding contemporary ageing the papers address three important dimensions of later old age. The first presentation by Gilleard directly addresses the corporeality of late old age noting its seeming undesirability and limitation. Gilleard posits that not only does the ageing body impact on the lived experiences of those in later old age but also acts as a cultural reference point for the representation of this period of the life course. Eliopoulos presents preliminary results from her qualitative study on social exclusion of individuals aged over 80 living in remote island environments of the Pacific Northwest. The research considers how such environments might, even in the absence of high levels of health and social care resources, mitigate some of the constraints associated with the ageing body. The chair, Paul Higgs will discuss the issue of ageism and how it is abstractly inscribed on the ageing body; often with little reference to the lived experiences of older people themselves. He will call for a more reflexive approach to ageism. Overall, the symposium seeks to draw gerontological attention to the complexities and possibilities surrounding the ageing body at later ages.

